# Online clinical reasoning skill training course for medical students: General medicine interest group

**DOI:** 10.1002/jgf2.498

**Published:** 2021-09-29

**Authors:** Kiyoshi Shikino, Mana Iwasaki, Ayaka Takahara, Naoki Kogayo, Shoichi Ito, Masatomi Ikusaka

**Affiliations:** ^1^ Department of General Medicine Chiba University Hospital Chiba Japan; ^2^ Chiba University School of Medicine Chiba Japan; ^3^ Matsudo City General Hospital Chiba Japan; ^4^ Department of Medical Education Graduate School of Medicine Chiba University Chiba Japan

**Keywords:** clinical reasoning, interest group, medical students, online

## CONFLICT OF INTEREST

The authors have stated explicitly that there are no conflicts of interest in connection with this article.

To the Editor

In Japan, the general medicine specialty began in April 2018. However, the number of general medicine senior residents was only 184 (2.2% of the total) in 2018, 180 (2.1%) in 2019, and 222 (2.4%) in 2020.^1^ Clinical diagnostic reasoning is cited as the factor with the strongest positive effect on medical students considering careers as general medicine physicians.^2^ Based on this evidence, the “General Medicine Interest Group (GMIG)” was launched at Chiba University School of Medicine in April 2017 to enable medical students to learn clinical reasoning skills. This face‐to‐face extracurricular activity, based on cooperation between students and general medicine specialists, is officially sanctioned by the Chiba University. GMIG aims to provide students participating in clinical clerkships with clinical diagnostic reasoning skills. Eighteen students participated in this 6‐month longitudinal course voluntarily.

However, owing to the COVID‐19 outbreak, actual face‐to‐face interaction became difficult to implement. To resolve the difficult situation, we tried educational activities that used an online meeting platform.

The educational sessions were held twice a month for 90 min each, from September 2020 to January 2021, using Zoom™. We had the participants learn the related symptomatology to prepare for the activities in advance (flipped classroom style).^3^ In the activity, online virtual medical interviews, an “impromptu” style of patient simulation encounter,^4^ were assigned. In the online interviews, a faculty member acted as the patient, and the student played the role of the doctor during a medical interview. The faculty provided clinical information in the response to the medical student's history taking. During the physical examination, the student asked the faculty for information about focused physical findings based on student's clinical hypothesis. In the response to the clinical hypothesis, the faculty presented them with the physical findings with real patient's photographs and videos (e.g., inspection of the pharynx, nystagmus, and deep tendon reflexes). Students from the same group could observe the interview, thereby promoting peer‐assisted learning.^4^ By using the chat function in the video conference system, the students could share the clinical reasoning process. Furthermore, they could hold small group discussions regarding the clinical reasoning process using a breakout room.

**FIGURE 1:**
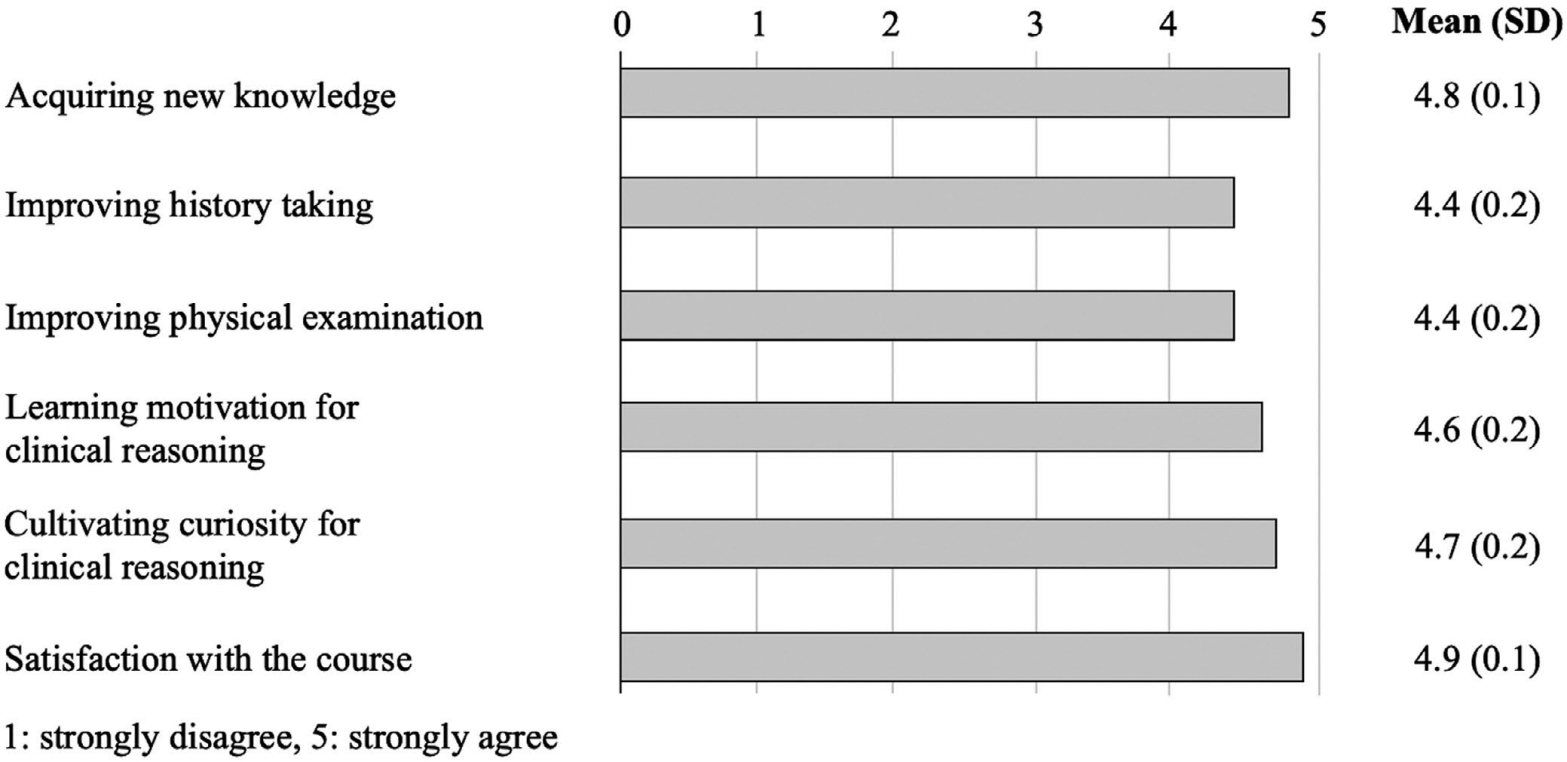
Results of the questionnaire survey after attending all nine sessions

Fourteen participants (10 males, four females, 78% response rate) responded to the questionnaire survey after attending all nine sessions. A five‐point Likert scale (1 = strongly disagree, 5 = strongly agree) was used in the questionnaire. The scores were 4.8 ± 0.1 for “acquiring new medical knowledge,” 4.4 ± 0.2 for “improving medical interviewing skills,” 4.4 ± 0.2 for “improving physical examination skills,” and 4.7 ± 0.2 for “cultivating curiosity for clinical reasoning” (Figure 1). The strengths of the online format were cited in the participants’ reflections, such as “Share and learn from abnormal findings in a timely manner.”

Even in limited circumstances, the effective use of an online meeting platform can contribute to improving students’ clinical reasoning skills. Educational seminars on clinical reasoning have proved effective for postgraduate physicians in Japan.^5^ Along the same lines, GMIG hopes to teach medical students about the clinical reasoning skills and grow curiosity about general medicine.
